# Supplementary material for: Nolz1 expression is required in dopaminergic axon guidance and striatal innervation
Source: Nat Commun. 2020 Jun 19;11:3111. doi: 10.1038/s41467-020-16947-6 (PMC7305235; doi:10.1038/s41467-020-16947-6)
Supplement: Supplementary file 1 — Supplementary Information [file 41467_2020_16947_MOESM1_ESM.pdf]

Supplementary information

Soleilhavoup et al., **Nolz1 expression is required in dopaminergic axon guidance and striatal innervation**

Supplementary figures 1-8

Supplementary tables 1-3

Supplementary Figure 1

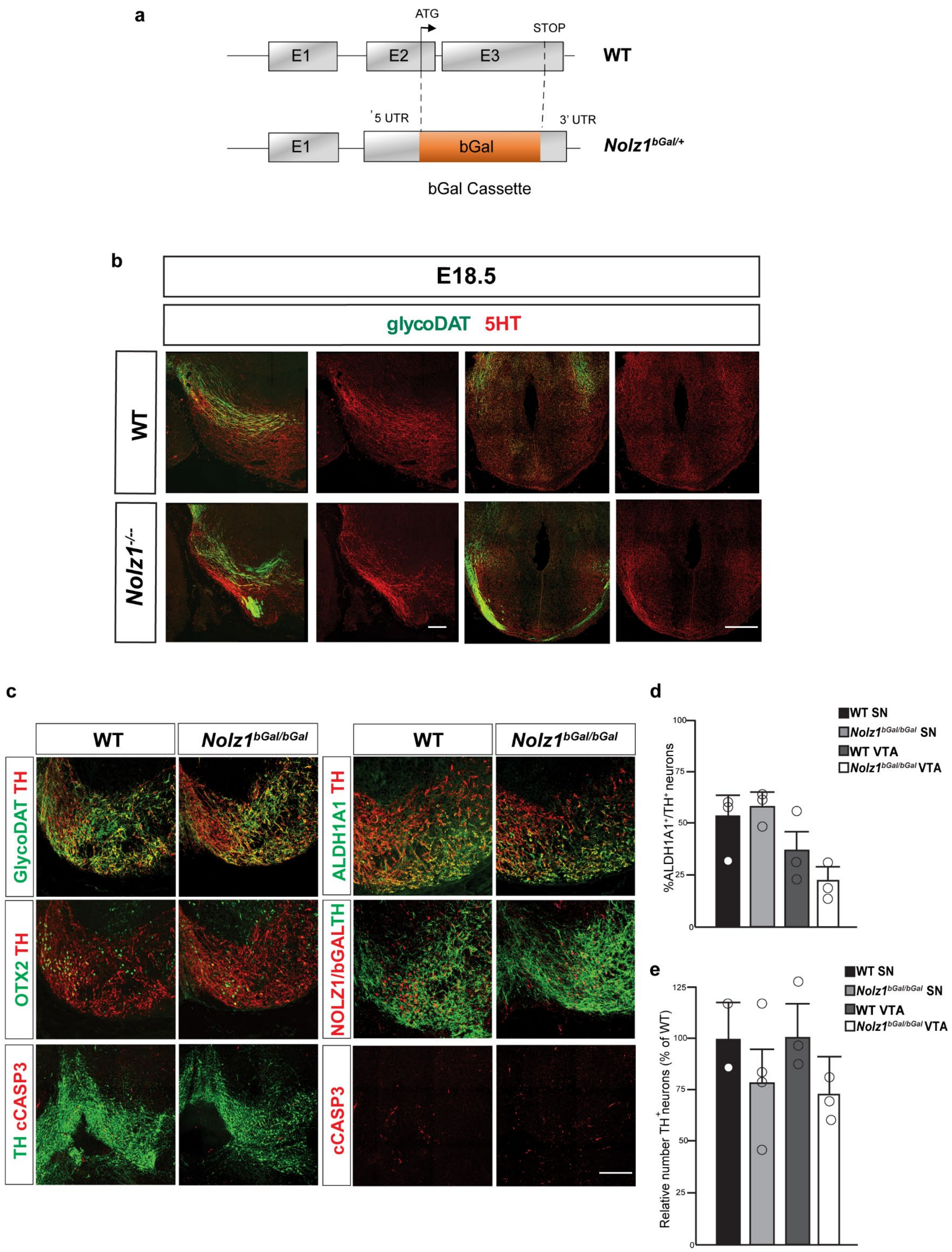

Supplementary Figure 1 . Analysis of DA subpopulation selective markers and serotonergic projections in WT and *Nolz1*$^{-/-}$ mutant embryos

(**a**) Schematic representation of the *Nolz1*$^{bGal/bGal}$ (also referred to as *Nolz1*$^{-/-}$) allele. Coding regions of exon 2 and exon 3 were replaced by *bGal*. (**b**) Immunofluorescence analysis of

GlycoDAT and 5HT expression on sagittal and coronal section of E18.5 Wt and *Nolz1*$^{-/-}$ mutant hypothalamus. Misguidance of serotonergic axons in direction of the midline in the *Nolz1*$^{-/-}$ mutant hypothalamus. **(c)** Immunostaining of DA markers on coronal sections of E18.5 mouse midbrain. **(d)** Percentage of ALDH1A1 positive TH expressing neurons in the SN and VTA of E18.5 Wt and *Nolz1*$^{-/-}$ mutant midbrain (n=3 biologically independent samples). **(e)** Graph shows the relative number of TH^+ neurons in the SN and VTA of E18.5 Wt and *Nolz1*$^{-/-}$ mutant midbrain. (n=3 biologically independent samples). Wt values are normalized to 100%. Mean values +/- standard deviation. Data are representative of 3 independent experiments (b, c). Scale bar: 200 µm (b), 500 µm (c). Source data are provided as a source data file.

Supplementary Figure 2

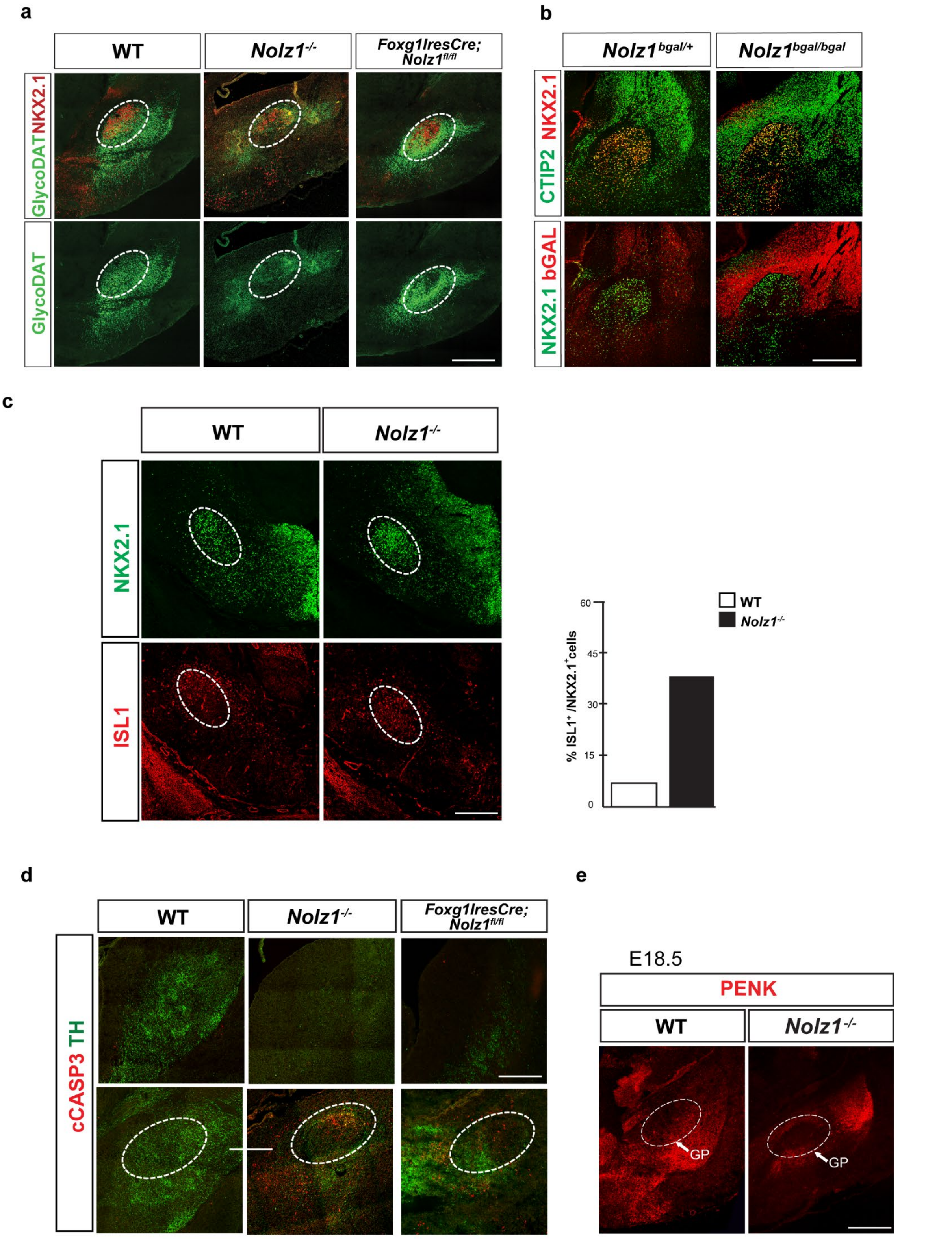

Supplementary Figure 2. Analysis of the globus pallidus development *Nolz1$^{-/-}$* mutant embryos

(**a**) Immunohistochemical analysis showing expression of NKX2.1 and GlycoDAT on coronal sections of the globus pallidus from E18.5 Wt, *Nolz1$^{-/-}$* and *Foxg1iresCre;Nolz1$^{fl/fl}$* mutant

embryos. Dashed circle indicates location of the globus pallidus. (**b**) Immunostaining of NKX2.1, CTIP2, and bGAL on coronal sections of the globus pallidus of E18.5 *Nolz1*$^{bGal/+}$ and *Nolz1*$^{bGal/bgal}$ mutant embryos. (**c**) Immunofluorescence analysis of NKX2.1 and ISL1 on coronal section of the globus pallidus of E13.5 WT and *Nolz1*$^{-/-}$ mutant embryos. Dashed circle indicates location of the globus pallidus. The percentage of ISL1^{+} against total NKX2.1^{+} cells in E18.5 Wt and *Nolz1*$^{-/-}$ mutant embryos. (**d**) Immunohistochemical analysis showing expression of cCASP3 and TH on coronal sections of the striatum of E18.5 Wt, *Nolz1*$^{-/-}$ and *Foxg1iresCre;Nolz1*$^{fl/fl}$ mutant embryos. (**e**) Immunohistochemical analysis of PENK expression in E18.5 Wt and *Nolz1*$^{-/-}$ mutant striatum. Dashed circle indicates location of the globus pallidus. Data are representative of 3 (a-e) independent experiments. Scale bar: 200 µm (a-e).

Supplementary Figure 3

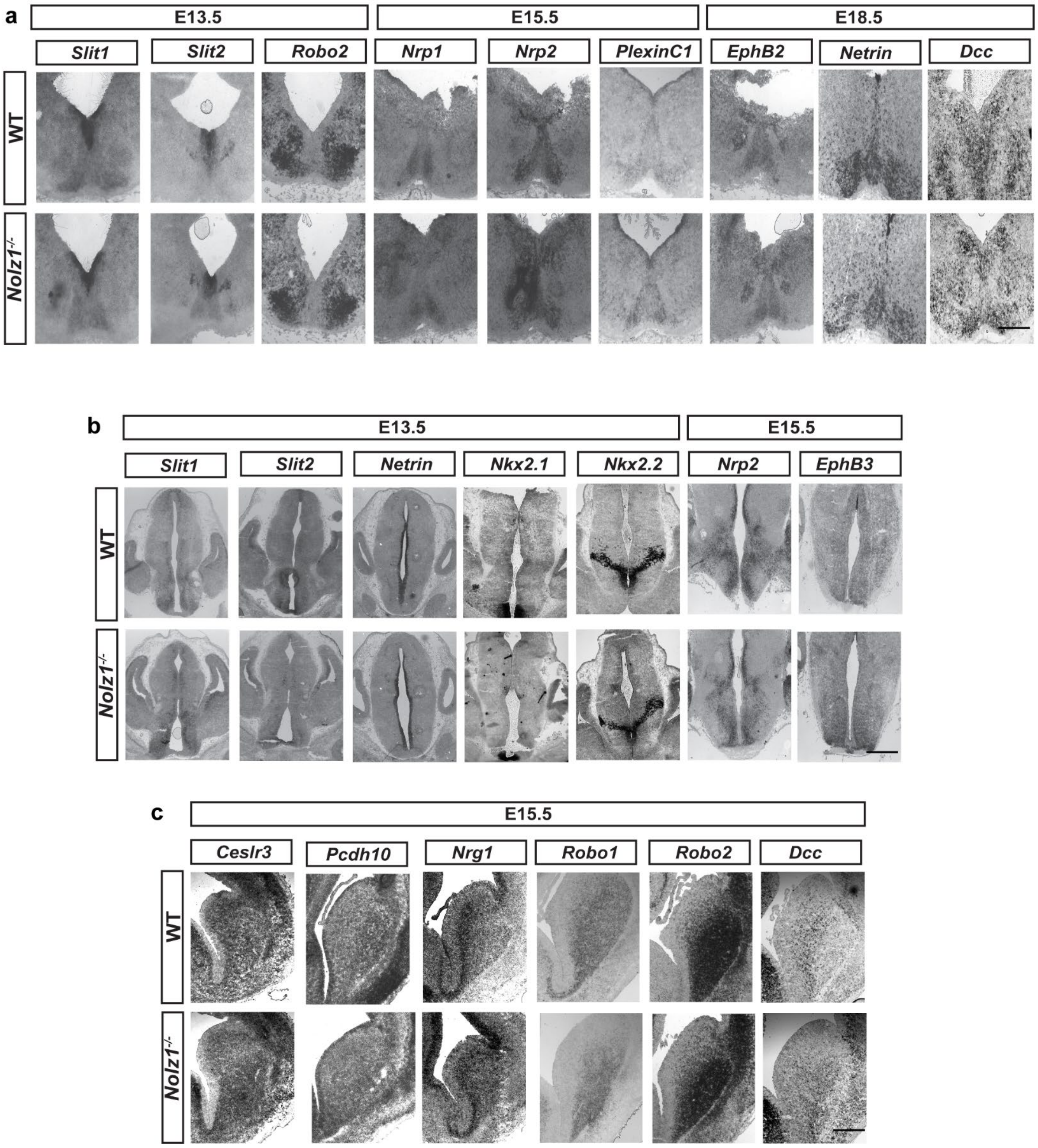

Supplementary Figure 3. Unaltered expression of genes involved in axon guidance in *Nolz1*$^{-/-}$ mutant embryos

(a) Analysis by in situ hybridization of several genes involved in axon guidance in Wt and *Nolz1*$^{-/-}$ mutant midbrain at E13.5, E15.5 and E18.5. Coronal sections. **(b)** Expression analysis of several genes involved in axon guidance in the diencephalon at E13.5 and E15.5 in Wt and *Nolz1*$^{-/-}$ mutant striatum. **(c)** In situ hybridisation for markers involved in striatal axon guidance at E15.5 in Wt and *Nolz1*$^{-/-}$ embryos. Data are representative of 3 (a-c) independent experiments Scale bar: 200 µm (a-c).

Supplementary Figure 4

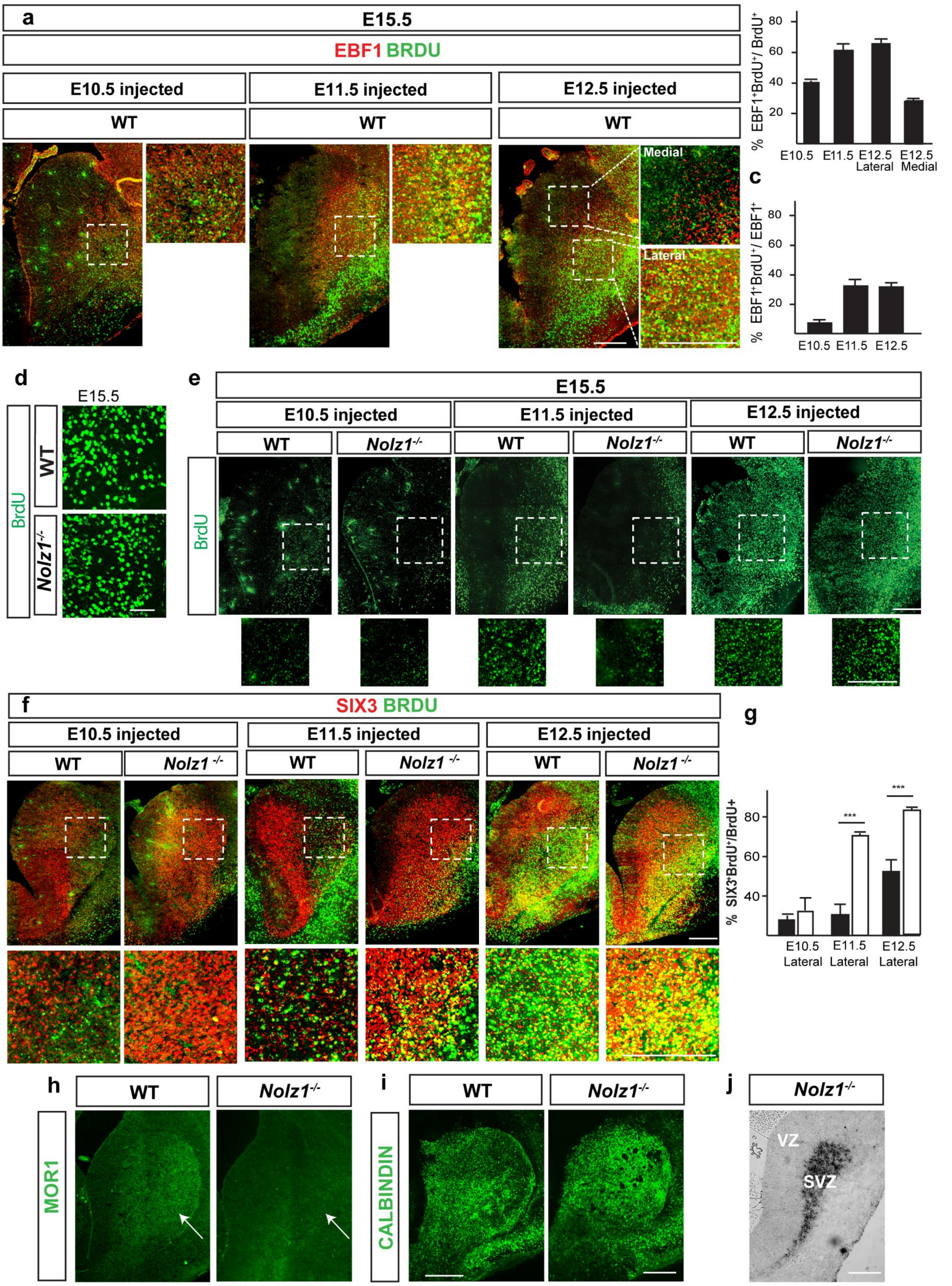

Supplementary Figure 4. Altered birthdate of striatal projection neurons in Nolz1$^{-/-}$ mutant striatum

(a) Immunofluorescence analysis of EBF1 and BrdU expression on coronal sections of E15.5 Wt striatum following BrdU injections at different time points (E10.5, E11.5 or E12.5). Dashed squares indicate magnified areas. **(b)** Graph showing the percentage of $EBF1^{+}BrdU^{+}$ double labelled cells against the total number of $BrdU^{+}$ cells in the E15.5 WT striatum following BrdU injection at different time points (E10.5, E11.5 or E12.5). Mean values +/- standard deviation; n=3 biologically independent samples. **(c)** Graph showing the percentage of $EBF1^{+}BrdU^{+}$ double labelled cells against the total number of $EBF1^{+}$ cells in the E15.5 Wt striatum following BrdU injection at different time points (E10.5, E11.5 or E12.5). Mean values +/- standard deviation; n=3 biologically independent samples. **(d)** BrdU expression visualized by immunofluorescence in the cortex of E15.5 Wt and *Nolz1*$^{-/-}$ mutant embryos injected with BrdU at E11.5. **(e)** Visualization of BrdU labelled cells by immunofluorescence in the striatum of E15.5 Wt and *Nolz1*$^{-/-}$ mutant embryos injected with BrdU at different time points (E10.5, E11.5 or E12.5). Coronal sections. Dashed squares indicate magnified areas. **(f)** Immunofluorescence analysis of SIX3 and BrdU expression on coronal sections of E15.5 Wt and *Nolz1*$^{-/-}$ mutant striatum following BrdU injections at different time points (E10.5, E11.5 or E12.5). Dashed squares indicate magnified areas. **(g)** Graph showing the percentage of $EBF1^{+}BrdU^{+}$ double labelled cells against the total number of $BrdU^{+}$ cells in the E15.5 Wt and *Nolz1*$^{-/-}$ mutant striatum following BrdU injection at different time points (E10.5, E11.5 or E12.5). Mean values +/- standard deviation; n=3 biologically independent samples; Two-sided, unpaired T-test: E11.5 Wt versus *Nolz1*$^{-/-}$ ***p=0.00134; E12.5 Wt versus *Nolz1*$^{-/-}$ ***p=0.0052. **(h-i)** Immunohistochemical analysis of MOR1 and CALBINDIN expression in the striatum of E18.5 Wt and *Nolz1*$^{-/-}$ mutant embryos. **(j)** *Nolz1* expression in E11.5 Wt striatum VZ (Ventricular zone), SVZ (Subventricular zone) (SVZ). Data are representative of 3 (d, e, h-j) independent experiments. Scale bar : 200 µm (a-i). Source data are provided as a source data file.

Supplementary Figure 5

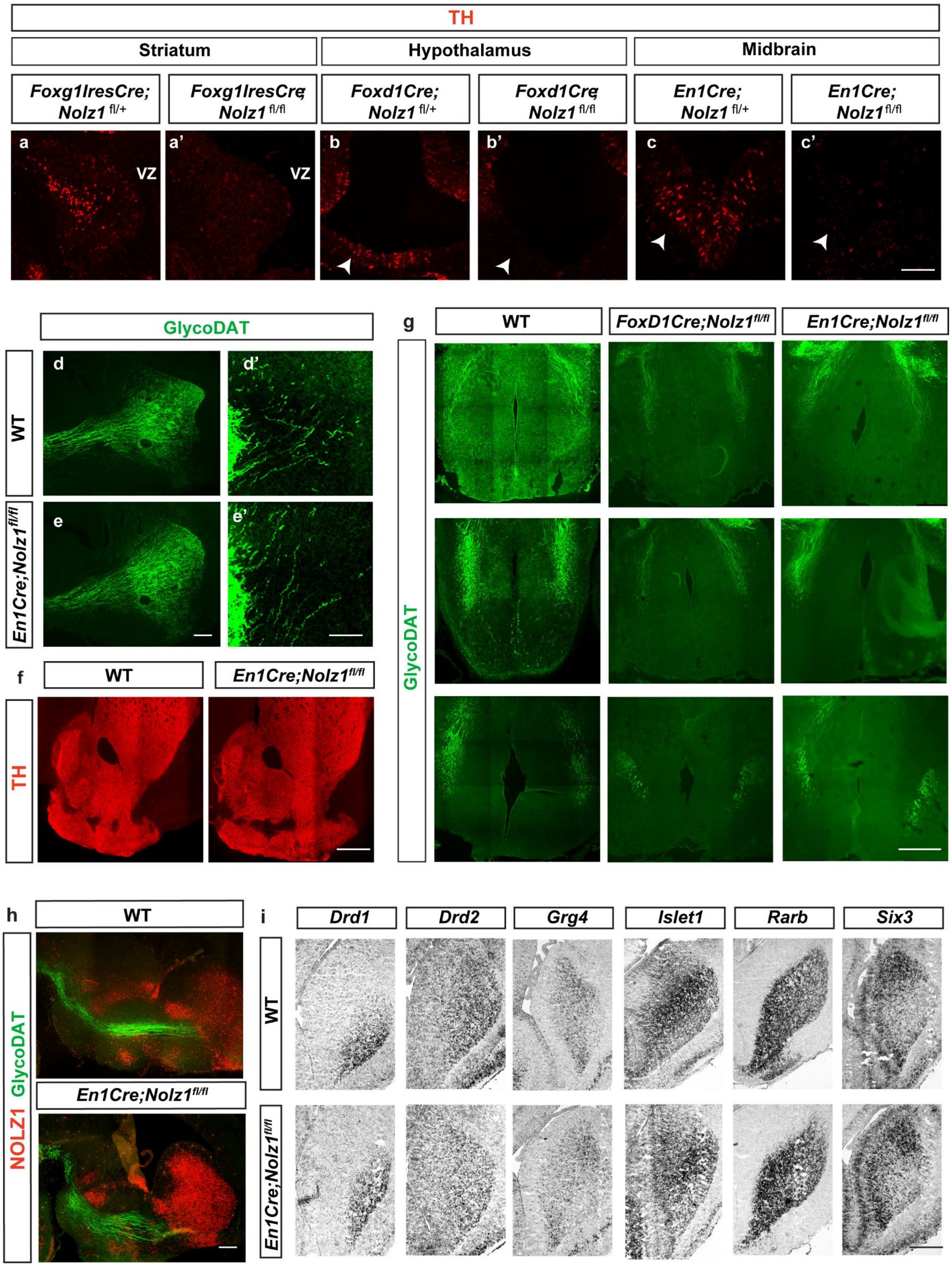

Supplementary Figure 5. Detailed characterization of Nolz1 conditional mouse lines

(a-c) Immunohistochemical analysis of NOLZ1 expression at E11.5 in the striatum, hypothalamus and midbrain of *Foxg1IresCre;Nolz1*$^{fl/fl}$, *Foxd1Cre;Nolz1*$^{fl/fl}$, and *En1Cre;Nolz1*$^{fl/fl}$ mutant embryos, respectively. **(d-e)** GlycoDAT staining in sagittal sections of E18.5 Wt and *En1Cre;Nolz1*$^{fl/fl}$ mutant brains shows normal initiation of PFC innervation. **(f)** TH

immunohistochemistry in the nucleus accumbens of 1-year-old Wt and *En1Cre;Nolz1*$^{fl/fl}$ mutant mice. **(g)** Immunohistochemical analysis of GlycoDAT positive fibres in the diencephalon of E18.5 Wt, *Foxd1Cre;Nolz1*$^{fl/fl}$, and *En1Cre;Nolz1*$^{fl/fl}$ mutant embryos reveals no difference in DA axonal projections. Coronal sections. **(h)** NOLZ1 and GlycoDAT immunostaining in E18.5 Wt and *En1Cre;Nolz1*$^{fl/fl}$ sagittal brain sections confirm no difference in DA axonal projections. **(i)** In situ hybridisation of several striatal markers in E15.5 Wt and *En1Cre;Nolz1*$^{fl/fl}$ mutant striatum. Data are representative of 3 (a-i) independent experiments. Scale bar: 100 µm (a-e; h-i), 50 µm (d', e'), 250 µm (f, g).

Supplementary Figure 6

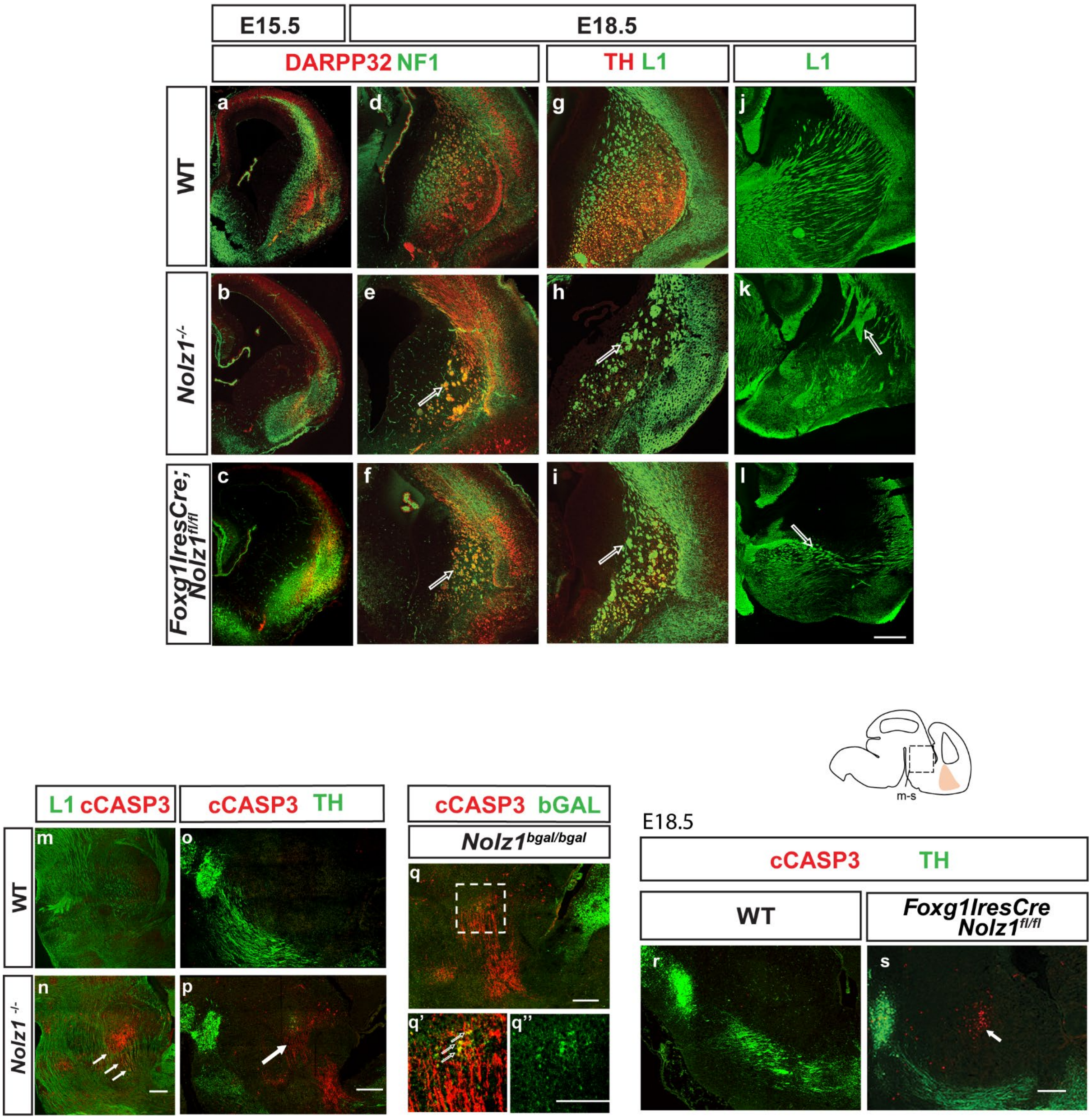

Supplementary Figure 6. Formation of forebrain axonal tracts is impaired in *Nolz1*$^{-/-}$ and *FoxG1-IRES-Cre; Nolz1*$^{fl/fl}$ mutant embryos

(a-f) Immunostaining for DARPP32 and NF1 on coronal sections of E15.5 and E18.5 Wt, *Nolz1*$^{-/-}$ and *FoxG1-IRES-Cre;Nolz1*$^{fl/fl}$ mutant striatum. Arrows (e-f) show abnormal fasciculation of

$NF1^+$ fibre bundles. **(g-i)** Immunostaining showing TH and L1 expression on coronal sections of E18.5 Wt, *Nolz1*$^{-/-}$ and *FoxG1-IRES-Cre;Nolz1*$^{fl/fl}$ mutant striatum. Arrows (H-I) show abnormal fasciculation of $L1^+$ fibre bundles. **(j-l)** Immunofluorescence labelling of L1 expression on sagittal sections of E18.5 Wt, *Nolz1*$^{-/-}$ and *FoxG1-IRES-Cre;Nolz1* $^{fl/fl}$ mutant striatum. (k, l) Arrows point to aberrant axon bundles. **(m-n)** Immunohistochemistry showing cCASP3 and L1 expression in thalamus of E18.5 Wt and *Nolz1*$^{-/-}$ mutant embryos on sagittal sections. (o-p) Immunostaining of cCASP3 and TH on sagittal sections of E18.5 Wt and *Nolz1*$^{-/-}$ mutant thalamus. **(q-q'')** Immunostaining showing the co-localisation of cCASP3 and bGAL in the thalamus of E18.5 *Nolz1*$^{bgal/bgal}$ mutant embryos. **(r-s)** TH and cCASP3 immunohistochemical analysis in E18.5 sagittal sections of Wt and *FoxG1iresCre;Nolz1*$^{fl/fl}$ mutant embryos. White arrow indicates cCASP3 expression. Data are representative of 3 (a-s) independent experiments. Scale bar: 200 µm (a-s).

Supplementary Figure 7

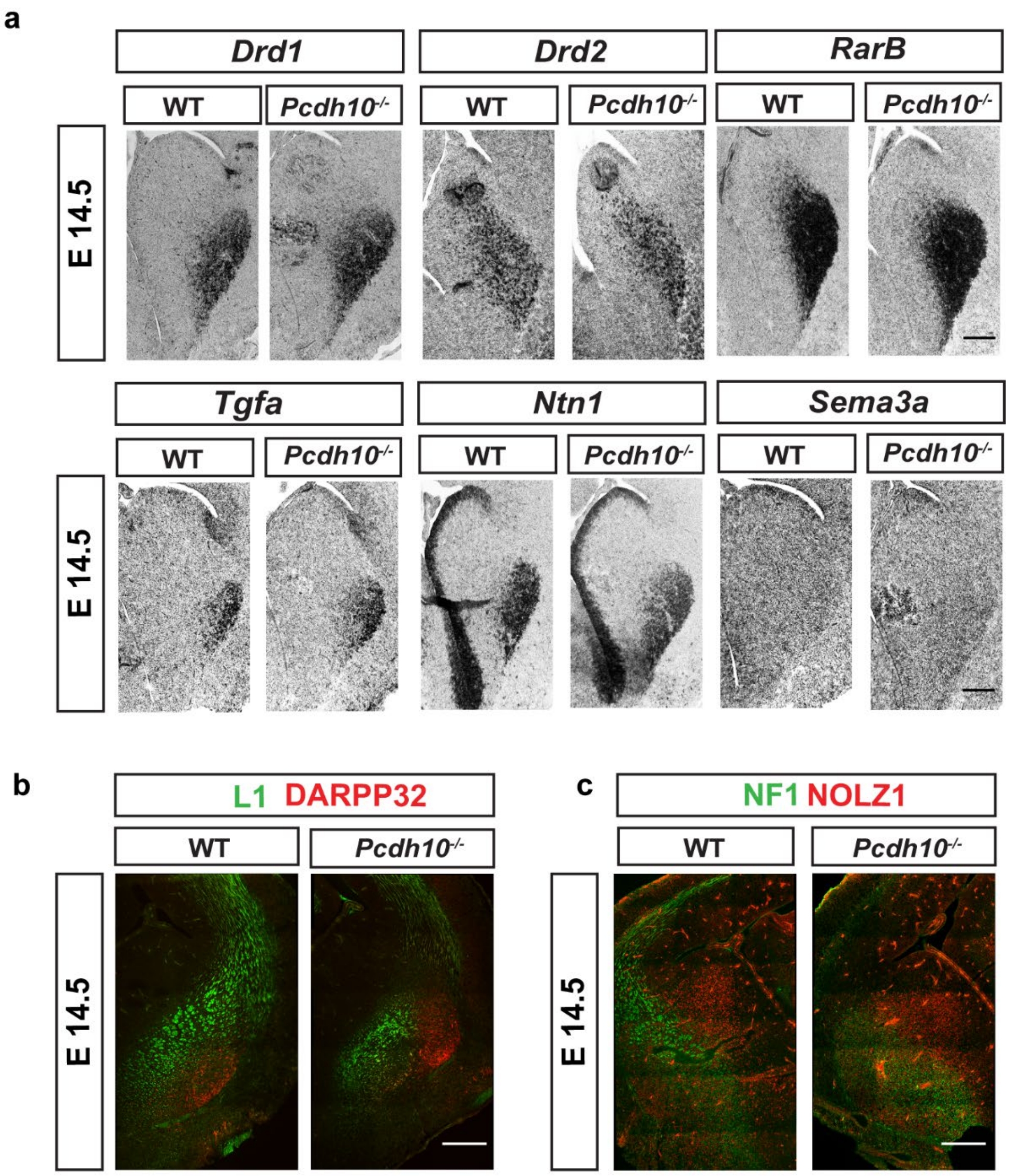

Supplementary Figure 7. Expression of several genes involved in axon guidance are unaltered in *Pcdh10*$^{-/-}$ mutant embryos.

(a) Expression analysis of several genes involved in striatal patterning and axon guidance by in situ hybridizations in E14.5 Wt and *Pcdh10*$^{-/-}$ mutant striatum. Coronal sections. **(b-c)** Immunohistochemical analysis showing expression of L1, DARPP32, NF1 and NOLZ1 on coronal sections of E14.5 Wt and *Nolz1*$^{-/-}$ mutant striatum. Data are representative of 3 (a-c) independent experiments. Scale bar: 200 µm (a-c).

Supplementary Figure 8

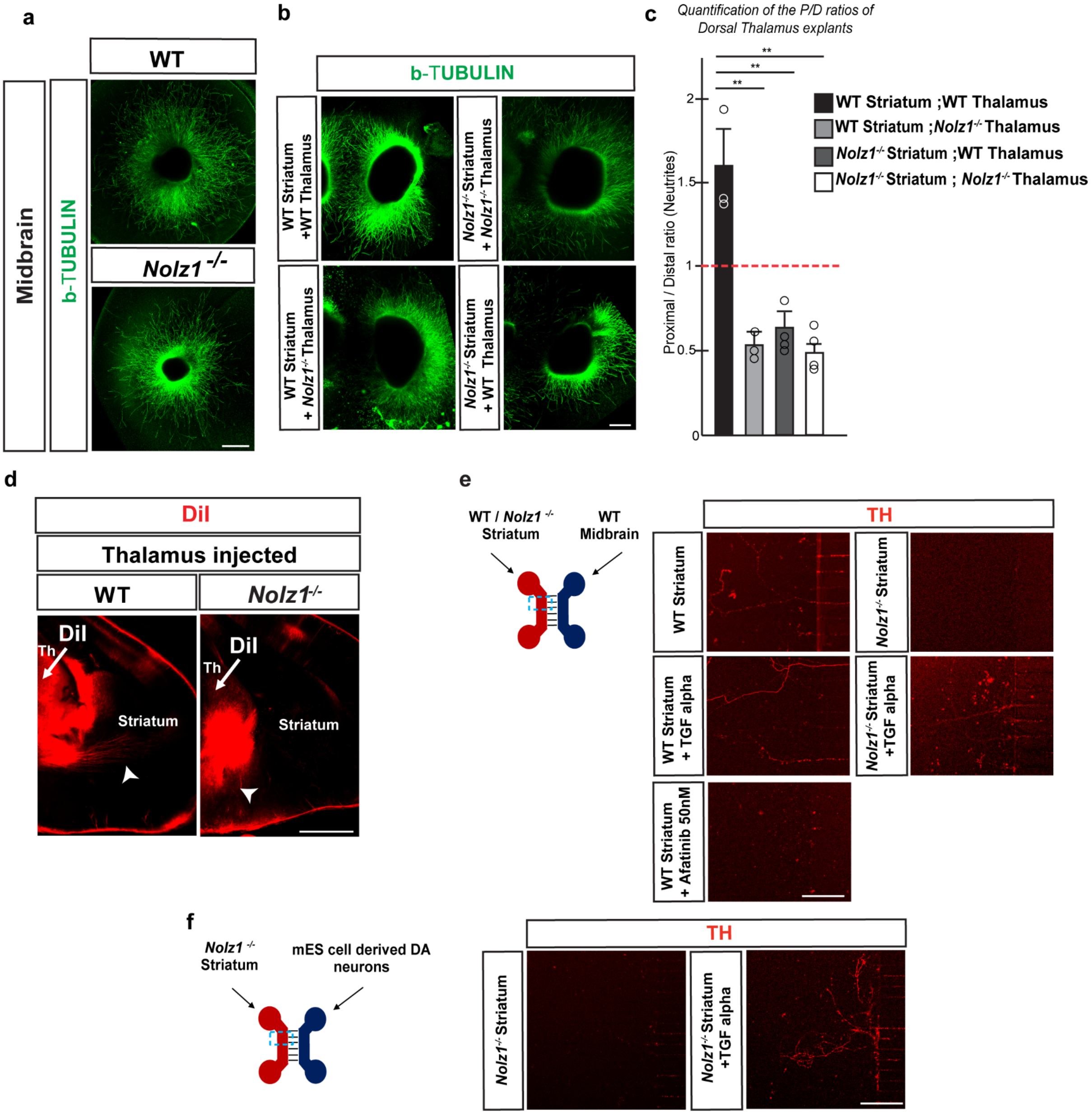

Supplementary Figure 8. *Nolz1$^{-/-}$* mutant striatum exhibits repulsive activities towards DA and thalamic axons. (a) Axonal outgrowth from E13.5 Wt and *Nolz1$^{-/-}$* mutant ventral midbrain explants visualized by b-TUBULIN. **(b)** Co-cultures of striatal and thalamic explants derived from E13.5 Wt and *Nolz1$^{-/-}$* mutant embryos and stained for b-TUBULIN. **(c)** The attractive and repulsive effects of Wt and *Nolz1$^{-/-}$* mutant striatum on the thalamic explants were quantified by measuring the axonal length in both the proximal (explant facing) and distal (facing away from the explant) compartment. Graph shows the proximal/distal ratio of the thalamic explants. Mean values +/- standard deviation; n=4 biologically independent experiments; Two-sided, unpaired T-test: Wt Striatum + Wt Thalamus versus *Nolz1$^{-/-}$* Striatum + *Nolz1$^{-/-}$* Thalamus **p= 0.00085; Wt Striatum + Wt Thalamus versus *Nolz1$^{-/-}$* Striatum + Wt Thalamus **p=0.0015; Wt Striatum + Wt Thalamus versus Wt Striatum + *Nolz1$^{-/-}$* Thalamus **p=0.0031. **(d)** DiI injection in the dorsal thalamus of E15.5 Wt and *Nolz1$^{-/-}$* mutant embryos.

Arrow points to the DiI injection site and arrowheads to thalamic axons. **(e)** Microfluidic assay to asses attractive and repulsive effects of Wt and *Nolz1*$^{-/-}$ mutant striatal tissue and TGFa signalling on DA axons. Axons were allowed to grow for 9 days and were labelled by TH. Primary DA neurons were cultured in the presence of either Wt striatal explants, *Nolz1*$^{-/-}$ mutant striatal explants, WT striatal explants with 50ng ml^{-1} TGFa, Nolz1$^{-/-}$ mutant striatal explants with 50ng ml^{-1} TGFa or Wt striatal explants with 50nM Afatinib. **(f)** mES cell-derived DA neurons were seeded in the cellular compartment of the microfluidics chamber. Striatal explants of E13.5 *Nolz1*$^{-/-}$ mutant embryos were added to the opposing well and cultured with or without 50ng ml^{-1} TGFa. Axons were allowed to grow for 9 days. Neurons were fixed and stained with TH (red). Data are representative of 3 (a, d, f) or 6 (e) independent experiments. Scale bar: 500 µm (a, b), 200 µm (d-f). Source data are provided as a source data file.

Supplementary Table 1

<u>Early patterning and neuronal specification</u>:
Lmx1a, Netrin1, Nkx2.1, Nkx2.2, Shh, Six3, Pax6

<u>Axonal pathfinding:</u>
Celsr3, Cxcl14, Dcc, EfnB3, EphA5, EphB2, FgfR2, Neogenin, Netrin1, Nrp1, Nrp2, plexin A1, plexin A2, PlexinC1, Robo1, Robo2, Robo3, Sema3a, Sema3f, Sema5b, Sema6a, Sema6c, Sema7a, Slit1, Slit2, Unc5C, Unc13C, Vegfc

<u>Dopaminergic neuron specific markers:</u>
Aldh1a1, Egfr, Erbb4, Lmx1a, Otx2, Sox6, Tgfa

<u>Hypothalamic neuron specific markers:</u>
Fezl1, Gad1, Isl1, Lim1, Meis2, Nkx2.1, Nkx2.2, Npy, Otp, Pmch, Pomc, Sf1, Six3, Sst

<u>Zona Incerta:</u>
Isl1, Lim1, pax6, Six3

Supplementary Table 1. List of genes that were normally expressed in *Nolz1*$^{-/-}$ mutant midbrain and diencephalon

Supplementary Table 2

Signalling pathway	Pathway component in the striatum	Receptor expression in DA neurons	Expression in Nolz1$^{-/-}$ mutant striatum (up/down)
BMP	Brinp1	Bmpr1b	DOWN
Netrin	Ntn1	Dcc	DOWN
RA	Rarb, Brinp1	Rara, Rarb, Rarg	DOWN
Chemokine	Cxcl14	Cxcr4	DOWN
Activin	Inhba	Tgfbr3	DOWN
Semaphorin	Sema3a	Nrp1, Nrp2, PlexinA2	DOWN
Tgf-alpha	Tgf-alpha	Egfr, Erbb4	DOWN
TNF	Fasl	Tnfrsf12a/19	UP
Shh	Shh	Boc	UP
VEGF	Vegfc	Kdr, Flt4	UP
WNT	Wnt4, Wnt5a	Lrp6, Fzd3	UP

Supplementary Table 2: **Signalling pathways altered in Nolz1$^{-/-}$ mutant striatum.** Differentially expressed pathway components between Wt and Nolz1$^{-/-}$ mutant striatum that were identified by RNA seq (See Supplementary data 1).

Supplementary Table 3. Resource table

Primary antibodies

Antigen	Species	Company (cat. no.)	Dilution
ALDH1a1	Rabbit	Sigma (HPA050139)	1:1000
B-GAL	Goat	AbD Serotec (4600-1409)	1:2000
BRDU	Rat	Abcam (ab6326)	1:500
CALBINDIN	Rabbit	Swant (CB38)	1:500
cCASP-3	Rabbit	Cell Signaling (9579S)	1:1000
CTIP2	Rat	Abcam (18465)	1:1000
DARPP32	Rabbit	Santa Cruz (sc-11365)	1:200
DAT	Rat	Merck Millipore (MAB369)	1:500
EBF1	Rabbit	Merck Millipore (ab10523)	1:500
FOXP1	Rabbit	Abcam (ab16645)	1:1000
FOXP2	Rabbit	Abcam (ab16046)	1:1000
GFP	Goat	Abcam (ab6673)	1:1000
ISL1	Mouse	DSHB (40.3A4)	1:50
L1	Rat	Merck Millipore (MAB5272)	1:1000
MOR1	Rabbit	Immunostar (24216)	1:4000
NF1	Mouse	DSHB (2H3-S1ea)	1:200
NKX2.1 (TTF1)	Rabbit	Abcam (ab76013)	1:1000
OTX2	Goat	R&D Systems (AF1979-SP)	1:500
SIX3	Guinea pig	Rockland (200-201-A26)	1:500
SIX3	Rabbit	Rockland (600-401-A26)	1:500
TAG1	Mouse	DSHB (4D7/TAG1-S)	1:200
TH	Sheep	Pel-Freez (P60101-150)	1:1000
TH	Rabbit	Pel-Freez (P40101-150)	1:1000 (IHC)<br>1:400 (iDISCO)
b-TUBULIN	Mouse	Biolegend (801202)	1:1000
ZNF503 (NOLZ1)	Rabbit	Atlas Antibodies (HPA026848)	1:1000

Secondary antibodies

Antigen	Species	Company (cat. no)	Dilution
Anti-Goat Alexa Fluor 488	Donkey	Invitrogen (A-11055)	1:500
Anti-Goat Alexa Fluor 555	Donkey	Invitrogen (A-21432)	1:500
Anti-Goat Alexa Fluor 647	Donkey	Invitrogen (A-21447)	1:500
Anti-Mouse Alexa Fluor 488	Donkey	Invitrogen (A-21202)	1:500
Anti-Mouse Alexa Fluor 555	Donkey	Invitrogen (A-31570)	1:500
Anti-Rabbit Alexa Fluor 488	Donkey	Invitrogen (A-21206)	1:500
Anti-Rabbit Alexa Fluor 555	Donkey	Invitrogen (A-31572)	1:500
Anti-Rabbit Alexa Fluor 647	Donkey	Invitrogen (A-32795)	1:500
Anti-Rat Alexa Fluor 488	Donkey	Invitrogen (A-21208)	1:500
Anti-Guinea pig Cy5	Donkey	Stratech (706-175-148-JIR)	1:800
Anti-Rat Alexa Fluor 488	Goat	Abcam (150157)	1:500

Primers used for in situ probe synthesis

Gene	Primer sequence	
Adam23	(Fwd) CTCAACACCAGGGTTGTCCT	(Rev) TCTTGCACTCGCCATTGTAG
Adora2a	(Fwd) ATGGGCTCCTCGGTGTACATCATG	(Rev) TCAGGAAGGGGCAAACTCTGAAGAC
Atxn1	(Fwd) CCCAAGCCCAGCAGACTA	(Rev) ATGACCAGCCCTGTCCAA
Brinp1	(Fwd) AACCACCAGCTGCCTAGAGA	(Rev) TAGCATTGGCTGTTTTGCAG
Cdh8	(Fwd) GATGGAGATGGGACAGCACT	(Rev) TGTTTTGCCAGAATGCTCAG
Chat	(Fwd) CAGCTGGCTTACTACAGGCTTT	(Rev) AATGAGGGGCTCTCTCTCTCTT
Chrm4	(Fwd) ATGGCGAACTTCACACCTGTCAATG	(Rev) CTACCTGGCTGTGCCGATGTTCC
Cxcl14	(Fwd) CTGCTCCTGCTGCTCCTG	(Rev) GTAGACCCTGCGCTTCTCGT
Drd1	(Fwd) TCCCAAGGAAGCTCCGAGAA	(Rev) AGGCTACCCAAATGTTACAA
Drd2	(Fwd) GAAGATCCTGCACTGCTGAGT	(Rev) ATGTTACAGAGTTGGAGCCCAG
Ebf1	(Fwd) TGACATGAGTCCCAGAGTGGAACTT	(Rev) CACTTCATTCTCCCCTTCCATAGCT
Epha8	(Fwd) CCTGTGAGCTGGGCTTCTAC	(Rev) TGAGTGTCAGGCAGATCCAG
EphB1	(Fwd) CAGTCGCTCCCCTTCAGA	(Rev) TGGCCACCAGAGACACAA
ER81	(Fwd) GTGCCTCTGTCTCACTTTGATG	(Rev) CTACTGGCCTGTGACTCAGTTG
ESR1	(Fwd) ATGACCCTTCACACCAAAGC	(Rev) TCATCATGCCCACTTCGTAA
Foxp1	(Fwd) ACCTTCCAAGTCCTCCCTAATC	(Rev) TAAATGGTGGTCTAACTTCCGC
Gad1	(Fwd) TGTGCCCAAACTGGTCCT	(Rev) TGGCCGATGATTCTGGTT
Gpr6	(Fwd) CGCTCAACGAGTCCCAAGTGGT	(Rev) TCAGACCTCACTGGGGGACCTG
Grg4	(Fwd) GTAAGCACTGGAAAGGACAACC	(Rev) GTTGCCCAAATAGACTCAAAGG
Grik3	(Fwd) CTCAATATCACTGAGGTTGCCA	(Rev) GGTGGAGATCTTGGATTTCTTG
LHX8	(Fwd) GAAGAGCGATCAGATGTTTGTG	(Rev) CACCTGTATGACACGTCTGCTT
Meis2	(Fwd) AGTCCAGAGGGTCAGCCC	(Rev) CTCCGCAGCATGGTTCTT
Netrin1	(Fwd) CTTCCTCACCGACCTCAATAAC	(Rev) TAGAGCTCCATGTTGAATCTGC
NPY1R	(Fwd) TCACCTTGGCTCTCGCTTAT	(Rev) TGATTCGCTTGGTCTCACTG
Nrp1	(Fwd) GGGAAGGTGACAAGAACATCTC	(Rev) CTTGTGTCTGTAGGTGACGCTC
Nrp2	(Fwd) GGTGAAGAATGGCTTCAGGTAG	(Rev) ATACTCCATGTCATAGCTGGGC
Pak3	(Fwd) TCATTGCACCAAGACCAGAG	(Rev) CCCACCATAGTGCTTCGTTT
Pcdh10	(Fwd) CACTTATCCTTATCATCGCCCT	(Rev) CAGATTTCAAAGAAGGCAGGAC
Pdyn	(Fwd) AGGAAAAGTTCAGGGGTCTCTC	(Rev) TCTCACAGTTCCCATGCAATAC
PlexinC1	(Fwd) AGGTTTTCCAAGCCTTCCTAAG	(Rev) CATGCAAGAGTTGTTTCTGAGC
PlexinD1	(Fwd) CCACTACAAGATACCTGAGGGC	(Rev) TGAGAGATGTGGGGAAGAAAC
PP2CA	(Fwd) CCTCTGCGAGAAGGCTAAAG	(Rev) CAGCTTGGTTACCACAACGA
PPP3CA	(Fwd) GGGACATCCATGGACAATTC	(Rev) CATGAAATTTGGGAGCCAGT
Ptprm	(Fwd) AGAGCTGGCCATCAGCAC	(Rev) AGTTCTTGGCTTTCCTTGCG
Rar Beta	(Fwd) GACCTTGAGGAACCAACAAAAG	(Rev) ACAACCTCGGTGTCTTGGTTAT
Rgs4	(Fwd) CCTGCGAACACAGTTCTTCA	(Rev) GAGACCAGGGAAGTGCAGTC
Robo1	(Fwd) CTGAAGACAACCAGAGAGGCTT	(Rev) CCTCCTTAAGTGGCTCTTCTGA
Robo2	(Fwd) GTAATAGGTGGCTTGTTCCCTG	(Rev) GCAGGATTTGTGTAGTGGCATA
Sema 3d	(Fwd) TGAGCAGATGTGGTACAAGGAG	(Rev) TAGATGTAGCCTGGTCCACAAA
Sema3a	(Fwd) TATCGAATTCGGCTTTGGAC	(Rev) CGCAGCAGTTCCAGAGTACA

Serpine2	(Fwd) TCCACGGTGATGCGATATAA	(Rev) CCTTTGATGGCTCAAACAT
Six3	(Fwd) AGAGTTGTCCATGTTCCAGTTG	(Rev) CTGATTTCGGTTTGTTCTAGGG
Sorl1	(Fwd) CGTGGTGAACCTTTTCTGGT	(Rev) CAGGTTCCAGCTTGCTTAGG
SP9	(Fwd) TACAAGTCGGGCTTCCACTC	(Rev) CCGTTGTGCGTCTTAATGTG
Srgap2	(Fwd) AGCCCGAGAACTGTCTTTCA	(Rev) AGTGGTTCCAAGGTGTCCAG
Tac1	(Fwd) CCCCTGAACGCACTATCTATTC	(Rev) CAGGAAACATGCTGCTAGGATA
Tgf alpha	(Fwd) CTCTGCTAGCGCTGGGTATC	(Rev) CAAGCAGTCCTTCCCTTCAG
TNN	(Fwd) CAAGACCTGGAACAGGGTGT	(Rev) TTCTGCCAGAGAAACGGTCT
Zfp 521	(Fwd) CAGACGCCAACAGCACAC	(Rev) TGGGCCGTATCCAGATGT

Specific reagents

Reagent name	Supplier	Cat number
**100mm tissue culture dishes**	Corning	3296
**24 well plates (mouse)**	Corning	3337
**96 well plates**	Greiner bio-one	655095
**Activin A**	R&D	338-AC
**Afatinib**	Selleckchem	BIB2992
**B27 supplement**	Life technologies	17504-044
**bFGF**	R&D	233-FB
**Collagen**	Gibco	A10483-01
**DAPI**	Sigma	D9564
**Denhart's**	Sigma	D2532
**Dig RNA**	Roche	11277073910
**Dimethyl sulfoxide (DMSO)**	Sigma	D8418
**DMEM high glucose**	Gibco	41965-039
**DMEM/F12**	Gibco	10565-018
**DMEM/F12 Glutamax**	Gibco	31331-028
**DPBS -Ca -Mg**	Gibco	14190-094
**Fetal bovine serum (FBS)**	Gibco	16141-079
**HBSS/Ca/Mg**	Gibco	14025-050
**Hepes**	Gibco	15630-049
**Insulin**	Gibco	12585-014
**KnockOut Serum Replacement (KSR)**	Gibco	10828028
**L-Glutamine**	Gibco	25030-024
**Laminin**	Sigma	L2020
**Levamisol**	Sigma	L9756-10G
**LIF**	Merck Millipore	ESG1107
**Lipofectamin LTX**	Invitrogen	15338-100
**N,N-Dimethylformamide**	Thermo Fisher	D/3840/08
**Netrin 1**	Adipogen	AG-40B-0040-C010

**Neurobasal**	Gibco	12348-017
**Non-adherent bacterial dishes**	Thermo Fisher	101R20
**Non-essential amino acids**	Gibco	11140-050
**Nunc- 24 well plates**	Thermo scientific	142475
**Nunc- 6 well plates**	Thermo scientific	140675
**Paraformaldehyde**	Sigma	P6148
**Penicillin-Streptomycin**	Thermo Fisher	15140-122
**Poly-D-Lysine**	Merck Millipore	A-003-E
**Poly-L-ornithine**	Sigma	P4957
**Progesterone**	Sigma	P6149
**Purmorphamine**	Calbiochem	540220-5
**Putrescine dihydrochloride**	Sigma	P5780
**Rnase Inhibitor**	Applied Biosystems	100021540
**RNeasy Micro kit**	Qiagen	74004
**SAG1.3**	Calbiochem	566660
**Sema3a (Fc chimera)**	R&D	1250-S3
**Sodium bicarbonate**	Sigma	S8761-100ml
**Sodium selenite**	Sigma	S9133
**Standard Neuron Device**	Xona Microfluidics	SND900
**Super Frost**	Thermo scientific	J1800AMNZ
**Sybr green Master Mix**	Applied Biosystems	4385612
**TGF alpha**	R&D	239-A
**Transferrin human**	Sigma	T8158
**TrypIE Express**	Gibco	12604-013
**β-Mercaptoethanol**	Sigma	M3148
