# Supplementary material for: Nolz1 expression is required in dopaminergic axon guidance and striatal innervation
Source: Nat Commun. 2020 Jun 19;11:3111. doi: 10.1038/s41467-020-16947-6 (PMC7305235; doi:10.1038/s41467-020-16947-6)
Supplement: Supplementary file 3 — Description of Additional Supplementary Files [file 41467_2020_16947_MOESM3_ESM.docx]

Description of Additional Supplementary Files

**Title:** Supplementary data 1.

**Description:** Differentially expressed genes between E18.5 Wt and Nolz1-/- mutant striatum. Differentially expressed genes identified by RNA sequencing (n=3 biologically independent samples). Log2 fold change (FC) and FC values of Nolz1-/- mutant striatum in comparison to Wt. Two-tailed Wald test was used to determine statistical significance. P-values were adjusted for multiple testing using the method of Benjamini and Hochberg and displayed as adjusted p-values (p-adj). Second tab shows the raw counts.

**Title:** Supplementary data 2.

**Description:** In situ validation of differentially expressed genes. Differentially expressed genes between Wt and Nolz1-/- mutant striatum (n=3 biologically independent samples) identified by RNA sequencing (see Supplementary data 1) that were validated by in situ hybridization. The fold change (FC) and statement about statistical significance of the genes that were validated by in situ are displayed in the table. For exact p-adj values see Supplementary data 1.
