# Supplementary material for: Nolz1 expression is required in dopaminergic axon guidance and striatal innervation
Source: Nat Commun. 2020 Jun 19;11:3111. doi: 10.1038/s41467-020-16947-6 (PMC7305235; doi:10.1038/s41467-020-16947-6)
Supplement: Supplementary file 6 — Reporting Summary [file 41467_2020_16947_MOESM6_ESM.pdf]

nature research

Corresponding author(s): Lia Panman

Last updated by author(s): May 22, 2020

Reporting Summary

Nature Research wishes to improve the reproducibility of the work that we publish. This form provides structure for consistency and transparency in reporting. For further information on Nature Research policies, see Authors & Referees and the Editorial Policy Checklist .

Statistics

For all statistical analyses, confirm that the following items are present in the figure legend, table legend, main text, or Methods section.

n/a	Confirmed	
☐	☒	The exact sample size (*n*) for each experimental group/condition, given as a discrete number and unit of measurement
☐	☒	A statement on whether measurements were taken from distinct samples or whether the same sample was measured repeatedly
☐	☒	The statistical test(s) used AND whether they are one- or two-sided<br>*Only common tests should be described solely by name; describe more complex techniques in the Methods section.*
☒	☐	A description of all covariates tested
☒	☐	A description of any assumptions or corrections, such as tests of normality and adjustment for multiple comparisons
☐	☒	A full description of the statistical parameters including central tendency (e.g. means) or other basic estimates (e.g. regression coefficient) AND variation (e.g. standard deviation) or associated estimates of uncertainty (e.g. confidence intervals)
☐	☒	For null hypothesis testing, the test statistic (e.g. *F*, *t*, *r*) with confidence intervals, effect sizes, degrees of freedom and *P* value noted<br>*Give P values as exact values whenever suitable.*
☒	☐	For Bayesian analysis, information on the choice of priors and Markov chain Monte Carlo settings
☒	☐	For hierarchical and complex designs, identification of the appropriate level for tests and full reporting of outcomes
☒	☐	Estimates of effect sizes (e.g. Cohen's *d*, Pearson's *r*), indicating how they were calculated

Our web collection on statistics for biologists contains articles on many of the points above.

Software and code

Policy information about availability of computer code

Data collection	Axiovert 200M inverted microscope, Zeiss LSM 880/LSM 510 confocal microscopes with Zeiss ZEN black 2012 as software and Ultramicrscope II (LaVision BioTec) lightsheet microscope with Imspector (version 5.0285.0) software (LaVision BioTec) were used for image acquisition. RNA sequencing was performed on the HiSeq4000. FastQC (version 0.11.5) was used for library verification. HISAT2 (version 2.1.0) was used to map the reads. Differentially expressed genes were identified using using the DESeq2 (version 1.18.1) package.
Data analysis	Adobe Photoshop CC2018 and Adobe Illustrator CC2018 were used for image processing . ImageJ and Excel 2016 were used to analyze the data

For manuscripts utilizing custom algorithms or software that are central to the research but not yet described in published literature, software must be made available to editors/reviewers. We strongly encourage code deposition in a community repository (e.g. GitHub). See the Nature Research guidelines for submitting code & software for further information.

Data

Policy information about availability of data

All manuscripts must include a data availability statement. This statement should provide the following information, where applicable:

- Accession codes, unique identifiers, or web links for publicly available datasets
- A list of figures that have associated raw data
- A description of any restrictions on data availability

The transcriptomic data have been deposited to ArrayExpress with the data set identifier E-MTAB-8240
The following figures have associated raw data: Fig. 2u, v, w; 3f; 4c, d, e; 5g; 6f, m, n; 7b, j; S1d, e; S2c; S4b, c, g; S8c
Data can be obtained from the corresponding author upon reasonable request

Field-specific reporting

Please select the one below that is the best fit for your research. If you are not sure, read the appropriate sections before making your selection.

[x] Life sciences [ ] Behavioural & social sciences [ ] Ecological, evolutionary & environmental sciences

For a reference copy of the document with all sections, see nature.com/documents/nr-reporting-summary-flat.pdf

Life sciences study design

All studies must disclose on these points even when the disclosure is negative.

Sample size	The sample size was not determined by using statistical methods. Required samples sizes were chosen based on experiences with similar experiments in previous publications. All data was collected from at least three independent experiments.
Data exclusions	No data was excluded from the analysis
Replication	All data was collected from at least three independent experiments (experiments were initiated at different days). All attempts at replication were successful
Randomization	Samples were allocated to different groups based on the genotype of the embryos/mice. Control and genetically altered embryos were from the same litter and analyzed in parallel to reduce variation.
Blinding	The investigators were not blinded when taking images of embryonic sections for phenotypic analysis as the genotypes were determined beforehand. However, all analysis that involved cell counting and measurements were done blinded. Furthermore, several people were involved in the analysis of the results, looked at the raw images and reproduced experimental outcomes independently. Also at least 3 independent replicates have been used.

Reporting for specific materials, systems and methods

We require information from authors about some types of materials, experimental systems and methods used in many studies. Here, indicate whether each material, system or method listed is relevant to your study. If you are not sure if a list item applies to your research, read the appropriate section before selecting a response.

Materials & experimental systems

n/a	Involved in the study	
[ ]	[x]	Antibodies
[ ]	[x]	Eukaryotic cell lines
[x]	[ ]	Palaeontology
[ ]	[x]	Animals and other organisms
[x]	[ ]	Human research participants
[x]	[ ]	Clinical data

Methods

n/a	Involved in the study	
[x]	[ ]	ChIP-seq
[x]	[ ]	Flow cytometry
[x]	[ ]	MRI-based neuroimaging

Antibodies

Antibodies used

Available in the Supplementary table 3

Validation

Antibodies were either used according to manufacturer's website or as validated in the literature. In some cases, further validation for use in frozen mouse brain sections was required.

- Aldh1a1 Rabbit Sigma (HPA050139), Literature: as validated for use in mouse sections by Wu et al., (2019).

- B-gal Goat AbD Serotec (4600-1409) Literature: as used by Blaess et al., (2011)

- Brdu Rat Abcam (ab6326) Literature: as validated for use in mouse sections by Cheng et al., (2019).

- Calbindin Rabbit Swant (CB38) Literature: as validated for use in mouse sections by Zhu et al., (2018).

- cCasp-3 Rabbit Cell signalling (9579S) Literature (as validated in Waldron et al., 2019)

- Ctip2 Rat Abcam (18465) Manufacturer statement: The application notes include recommended starting dilutions; optimal dilutions/concentrations should be determined by the end user (https://www.abcam.com/ctip2-antibody-25b6-chip-grade-ab18465.html). Upon validation, we used 1:1000 dilution as we observed optimal results for this concentration.

- Darpp-32 Rabbit Santa Cruz (sc-11365) Literature: validated for use in frozen mouse sections at 1:1000 in Garcia-Miralles et al. (2017). Upon validation we used 1:200 for optimal results.

- DAT Rat Merck Millipore (MAB369) Literature: as validated for use in frozen mouse sections by Panman et al. (2014)

- EBF1 Rabbit Merck Millipore (ab10523) Manufacturer statement: Immunohistochemistry Analysis: A 1:500 dilution from a representative lot detected EBF-1 in mouse cryosections of wild type spinal cord tissue (https://www.merckmillipore.com/GB/en/product/Anti-EBF-1-Antibody,MM_NF-AB10523).

- Foxp1 Rabbit Abcam (ab16645) Literature: as validated for use in mouse cells by Krausher et al. (2015)

- Foxp2 Rabbit Abcam (ab16046) Literature: as validated for use in mouse brain sections by Campbell et al. (2009)

- GFP Goat Abcam (ab6673) Manufacturer statement: The application notes include recommended starting dilutions; optimal dilutions/concentrations should be determined by the end user.(https://www.abcam.com/gfp-antibody-ab6673.html?productWallTab=ShowAll) Manufacturer recommended 1:200 – 1:1000 as optimal dilutions. Upon validation, we used 1:1000 as we observed optimal results for this concentration.

- Isl1 Mouse DSHB (40.3A4) Literature: as validated for use in frozen mouse sections by Cambier et al. (2014).

- L1 Rat Merck Millipore (MAB5272) Manufacturer statement: This Anti-Neural Cell Adhesion Molecule L1 Antibody, clone 324 is validated for use in IC, IH, WB for the detection of Neural Cell Adhesion Molecule L1. Optimal working dilutions must be determined by end user (https://www.merckmillipore.com/GB/en/product/Anti-Neural-Cell-Adhesion-Molecule-L1-Antibody-clone-324,MM_NF-MAB5272). Upon testing various concentrations, we obtained optimal results at 1:1000.

-MOR1 Rabbit Immunostar (24216). Literature: as validated for use in mouse tissue by Kelly et al., (2018) Neuron. We obtained similar results when using it at a dilution of 1:4000.

- NF1 Mouse DSHB (2H3-S1ea) Literature: as validated for use in mouse embryonic tissue by Seibt et al. (2003). Upon further validation, we observed optimal results at 1:200.

- NKX2.1 Rabbit Abcam (ab76013) Manufacturer statement: The application notes include recommended starting dilutions; optimal dilutions/concentrations should be determined by the end user (https://www.abcam.com/ttf1-antibody-ep1584y-ab76013.html). Upon validation for use in mouse brain sections, we observed optimal results at 1:1000.

- Otx2 Goat R&D Systems (AF1979-SP) Literature: as validated for use in mouse frozen sections by Chen et al. (2019). Upon further validation, we observed optimal results at 1:500.

- Six3 Guinea Pig Rockland (200-201-A26) Literature: as validated for use in paraffin embedded mouse brain section by Madrigal et al. (2016). Upon further validation we observed optimal results at 1:500 for use in frozen mouse brain sections.

- Six3 Rabbit Rockland (600-401-A26) Literature: as validated for use in frozen mouse sections by Xu et al. (2018).

- TAG1 Mouse DSHB (4D7/TAG1-S) Literature: as validated for use in mouse brain sections by Morante-Oria et al (2003). Upon further validation in frozen mouse section, we observed optimal results at 1:200.

- TH Sheep Pel-Freez (P60101-150) Manufacturer statement: IF 1:1000 (https://www.pelfreez-bio.com/wp-content/uploads/2014/07/74041-PDS-P60101-Tyrosine-Hydroxylase-Antibody-Sheep-Rev-02.pdf). Used according to manufacturer's instructions.

- TH Rabbit Pel-Freez (P40101-150) Manufacturer statement: IF 1:1000 (https://www.pelfreez-bio.com/wp-content/uploads/2014/07/74075-PDS-P40101-Tyrosine-Hydroxylase-Antibody-Rabbit-Rev-02.pdf). Used according to manufacturer's instructions.

- b-Tubulin Mouse Biolegend (801202 Manufacturer statement: IF 1:1000 (https://www.biolegend.com/en-us/products/purified-anti-beta-tubulin-antibody-11242). Used according to manufacturer's instructions.

- Znf503 Rabbit Atlas Antibodies (HPA026848) Literature: as validated for use in frozen mouse section by Panman et al., (2014)

Relevant Citations:

Blaess S, Bodea GO, Kabanova A, Chanet S, Mugniery E, Derouiche A, Stephen D, Joyner AL. Temporal-spatial changes in Sonic Hedgehog expression and signaling reveal different potentials of ventral mesencephalic progenitors to populate distinct ventral midbrain nuclei. Neural Dev. 2011 Jun 20;6:29. doi: 10.1186/1749-8104-6-29.

Campbell P, Reep RL, Stoll ML, Ophir AG, Phelps SM. Conservation and diversity of Foxp2 expression in muroid rodents:

functional implications. J Comp Neurol. 2009;512(1):84-100. doi:10.1002/cne.21881

Cambier L, Plate M, Sucov HM, Pashmforoush M. Nkx2-5 regulates cardiac growth through modulation of Wnt signaling by R-spondin3. Development. 2014;141(15):2959-2971. doi:10.1242/dev.103416

Chen Y, Yang J, Geng H, et al. Photoreceptor degeneration in microphthalmia (Mitf) mice: partial rescue by pigment epithelium-derived factor. Dis Model Mech. 2019;12(1):dmm035642. Published 2019 Jan 11. doi:10.1242/dmm.035642

Cheng, X., Yeung, P.K.K., Zhong, K. et al. Astrocytic endothelin-1 overexpression promotes neural progenitor cells proliferation and differentiation into astrocytes via the Jak2/Stat3 pathway after stroke. J Neuroinflammation 16, 227 (2019).

Garcia-Miralles M, Geva M, Tan JY, et al. Early pridopidine treatment improves behavioral and transcriptional deficits in YAC128 Huntington disease mice. JCI Insight. 2017;2(23):e95665. Published 2017 Dec 7. doi:10.1172/jci.insight.95665

Kelly SM, Raudales R, He M, Lee JH, Kim Y, Gibb LG, Wu P, Matho K, Osten P, Graybiel AM, Huang ZJ. Radial Glial Lineage Progression and Differential Intermediate Progenitor Amplification Underlie Striatal Compartments and Circuit Organization. Neuron 2018 Jul 25;99(2):345-361.e4. doi: 10.1016/j.neuron.2018.06.021

Kraushar M.L., Viljetic B., Wijeratne S.H.R., Thompson K., Jiao X., Pike J.W., Medvedeva V., Groszer M., Kiledjian M., Hart R.P., Rasin M.R., (2015). Thalamic WNT3 Secretion Spatiotemporally Regulates the Neocortical Ribosome Signature and mRNA Translation to Specify Neocortical Cell Subtypes, Journal of Neuroscience, 35 (31) 10911-10926; DOI: 10.1523/ JNEUROSCI.0601-15.2015

Madrigal MP, Moreno-Bravo JA, Martínez-López JE, Martínez S, Puelles E. Mesencephalic origin of the rostral Substantia nigra pars reticulata. Brain Struct Funct. 2016;221(3):1403-1412. doi:10.1007/s00429-014-0980-9

Morante-Oria J, Carleton A, Ortino B, Kremer EJ, Fairen A, Lledo PM. 2003. Subpallial origin of a population of projecting pioneer neurons during corticogenesis. Proc Natl Acad Sci U S A. 100: 12468--12473.

Panman L., et al .Sox6 and Otx2 control the specification of substantia nigra and ventral tegmental area dopamine neurons, Cell Rep., 8 (2014), pp. 1018-1025

Seibt J, Schuurmans C, Gradwhol G, Dehay C, Vanderhaeghen P, Guillemot F, Polleux F (2003) Neurogenin2 specifies the connectivity of thalamic neurons by controlling axon responsiveness to intermediate target cues. Neuron 39:439 – 452.

Zhu F, Cizeron M, Qiu Z, Benavides Piccione R, Kopanitsa M, Skene N, et al. Architecture of the Mouse Brain Synaptome. Neuron. 2018;99:781

Waldron A., Wilcox C., Francklyn C., Ebert A., (2019). Knock-Down of Histidyl-tRNA Synthetase Causes Cell Cycle Arrest and Apoptosis of Neuronal Progenitor Cells in vivo, Front. Cell Dev. Biol., 7:67. doi: 10.3389/fcell.2019.00067

Wu J, Kung J, Dong J, et al. Distinct Connectivity and Functionality of Aldehyde Dehydrogenase 1a1-Positive Nigrostriatal Dopaminergic Neurons in Motor Learning. Cell Rep. 2019;28(5):1167-1181.e7. doi:10.1016/j.celrep.2019.06.095

Xu Z, Liang Q, Song X, et al. SP8 and SP9 coordinately promote D2-type medium spiny neuron production by activating Six3 expression. Development. 2018;145(14):dev165456. Published 2018 Jul 23. doi:10.1242/dev.165456

Eukaryotic cell lines

Policy information about cell lines

Cell line source(s)	Nes-Lmx1a mouse ES cell line
Authentication	Line was karyotyped
Mycoplasma contamination	Line was tested negatively for mycoplasma
Commonly misidentified lines (See ICLAC register)	No commonly misidentified lines were used

Animals and other organisms

Policy information about studies involving animals; ARRIVE guidelines recommended for reporting animal research

Laboratory animals	Mice. Nolz1+/-, Nolz1fl/+, FoxG1IresCre, FoxD1Cre, En1Cre and Pcdh10+/- mouse lines. Males and females between 2 months and 6 months old were used in breeding couples to obtain embryos. For the analysis we used embryos of different stages as

indicated in the figure legends. The sex of the embryos used in this study was not determined.

Wild animals	No wild animals were used.
Field-collected samples	This study did not use field-collected samples
Ethics oversight	Ethical permission was obtained by the AWERB of the University of Leicester

Note that full information on the approval of the study protocol must also be provided in the manuscript.
